# Supplementary material for: paramix: An R package for parameter discretisation in compartmental models, with application to calculating years of life lost
Source: PLoS Comput Biol. 2025 Sep 8;21(9):e1013420. doi: 10.1371/journal.pcbi.1013420 (PMC12425178; doi:10.1371/journal.pcbi.1013420)
Supplement: S1 Text — (PDF) [file pcbi.1013420.s001.pdf]

Supplementary Information File
paramix: An R package for parameter discretisation in compartmental models, with application to calculating years of life lost

Section 1. *paramix* functions

```r
#' @keywords internal
vec_integrate <- function(f, lowers, uppers, ...) {
  integrals <- numeric(length(lowers))
  for (i in seq_along(lowers)) {
    integrals[i] <- integrate(f, lowers[i], uppers[i], ...)$value
  }
  return(integrals)
}

#' @title Create the Blending and Distilling Object
#'
#' @description
#' Based on model and output partitions, create a mixing partition and
#' associated weights. That table of mixing values can be used to properly
#' discretize a continuously varying (or otherwise high resolution) parameter to
#' a relatively low resolution compartmental stratification, and then
#' subsequently allocate the low-resolution model outcomes into the most likely
#' high-resolution output partitions.
#'
#' @param f_param a function, `f(x)` which transforms the feature (e.g. age),
#' to yield the parameter values. Alternatively, a `data.frame` where the first
#' column is the feature and the second is the parameter; see [xy.coords()] for
#' details. If the latter, combined with `pars_interp_opts` to create a
#' parameter function.
#'
#' @param f_pop like `f_param`, either a density function (though it does
#' not have to integrate to 1 like a pdf) or a `data.frame` of values. If the
#' latter, it is treated as a series of populations within intervals, and
#' then interpolated with `pop_interp_opts` to create a density function.
#'
#' @param model_partition a numeric vector of cut points, which define the
#' partitioning that will be used in the model; must be length > 1
#'
#' @param output_partition the partition of the underlying feature; must be
#' length > 1
#'
#' @param pars_interp_opts a list, minimally with an element `fun`,
#' corresponding to an interpolation function. Defaults to [splinefun()]
#' "natural" interpolation.
#'
#' @param pop_interp_opts like `pars_interp_opts`, but for density. Defaults to
#' [approxfun()] "constant" interpolation.
#'
#' @details
#' The `alembic` function creates a mixing table, which governs the conversion
#' between model and output partitions. The mixing table a [data.table()] where
#' each row corresponds to a mixing partition \eqn{c_i}, which is the union of
#' the model and output partitions - i.e. each unique boundary is included.
#' Within each row, there is a `weight` and `relpop` entry, corresponding to
#'
#' \deqn{
#' \textrm{weight}_i = \int_{c_i} f(x)\rho(x)\text{d}x
#' }
#'
```

```r
#' \deqn{
#' \textrm{relpop}_i = \int_{c_i} \rho(x)\text{d}x
#' }
#'
#' where \eqn{f(x)} corresponds to the `f_param` argument and \eqn{\rho(x)}
#' corresponds to the `f_pop` argument.
#'
#' This mixing table is used in the [blend()] and [distill()] functions.
#'
#' When `blend`ing, the appropriately weighted parameter for a model partition
#' is the sum of \eqn{\textrm{weight}_i} divided by the \eqn{\textrm{relpop}_i}
#' associated with mixing partition(s) in that model partition. This corresponds
#' to the properly, population weighted average of that parameter over the
#' partition.
#'
#' When `distill`ing, model outcomes associated with weighted parameter from
#' partition \eqn{j} are distributed to the output partition \eqn{i} by the sum
#' of weights in mixing partitions in both \eqn{j} and \eqn{i} divided by the
#' total weight in \eqn{j}. This corresponds to proportional allocation
#' according to Bayes rule: the outcome in the model partition was relatively
#' more likely in the higher weight mixing partition.
#'
#' @return a `data.table` with columns: `model_partition`, `output_partition`, `weight` and
#' `relpop`. The first two columns identify partition lower bounds, for both the model
#' and output, the other values are associated with; the combination of
#' `model_partition` and `output_partition` forms a unique identifier, but individually they
#' may appear multiple times. Generally, this object is only useful as an input
#' to the [blend()] and [distill()] tools.
#'
#' @examples
#' ifr_levin <- function(age_in_years) {
#'   (10^(-3.27 + 0.0524 * age_in_years))/100
#' }
#' age_limits <- c(seq(0, 69, by = 5), 70, 80, 101)
#' age_pyramid <- data.frame(
#'   from = 0:101, weight = ifelse(0:101 < 65, 1, .99^(0:101-64))
#' )
#' age_pyramid$weight[102] <- 0
#' # flat age distribution, then 1% annual deaths, no one lives past 101
#' ifr_alembic <- alembic(ifr_levin, age_pyramid, age_limits, 0:101)
#'
#' @seealso [blend()]
#' @seealso [distill()]
#' @importFrom utils head tail
#' @importFrom stats integrate
#' @import data.table
#' @export
alembic <- function(
    f_param,
    f_pop,
    model_partition,
    output_partition,
    pars_interp_opts = interpolate_opts(
      fun = stats::splinefun, kind = "point",
      method = "natural"
    ),
    pop_interp_opts = interpolate_opts(
      fun = stats::approxfun, kind = "integral",
      method = "constant", yleft = 0, yright = 0
    )
```

```r
) {

  # create the mixing partition: this the union of the model and output
  # partitions. We define an internal function here which also does a variety
  # of error handling, but ultimately provides the union of these inputs when
  # there are no errors
  mixing_partition <- make_partition(model_partition, output_partition)

  # get the boundaries for the mixing partition intervals
  c_i <- head(mixing_partition, -1)
  c_iplus1 <- tail(mixing_partition, -1)

  # guarantee that `f_param` and `f_pop` are proper functions; if they are
  # provided as a table of values, conversion will occur via the specified
  # interpolation options
  f_param <- to_function(f_param, range(mixing_partition), pars_interp_opts)
  f_pop <- to_function(f_pop, range(mixing_partition), pop_interp_opts)

  # create the weight function, which is f_param(x)*f_pop(x)
  f <- make_weight(f_param, f_pop)

  # for each mixing interval, calculate:
  # the weight i = integral_{c i}^{c i+1} param(x)*density(x) dx
  # the population i = integral_{c i}^{c i+1} density(x) dx
  # `vec_integrate` wraps stats::integrate to vectorize lower and upper args
  weight <- vec_integrate(f, c_i, c_iplus1, subdivisions = 1000L)
  relpop <- vec_integrate(f_pop, c_i, c_iplus1, subdivisions = 1000L)

  # get which model and output partitions the c_i correspond to
  which_model_part <- findInterval(c_i, model_partition)
  which_out_part <- findInterval(c_i, output_partition)

  # create the mixing table
  ret <- data.table(
    model_partition = model_partition[which_model_part],
    output_partition = output_partition[which_out_part],
    weight = weight, relpop = relpop
  )

  # capture the functional forms with the mixing table
  setattr(ret, "f_param", f_param)
  setattr(ret, "f_pop", f_pop)

  return(ret)

}

#' @title Blend Parameters
#'
#' @description
#' `blend` extracts aggregate parameters from an `alembic` object.
#'
#' @param alembic_dt an [alembic()] return value
#'
#' @return a `data.table` of with two columns: `model_partition` (partition lower
#' bounds) and `value` (parameter values for those partitions)
#'
#' @examples
#' ifr_levin <- function(age_in_years) {
#'   (10^(-3.27 + 0.0524 * age_in_years))/100
```

```r
#' }
#'
#' age_limits <- c(seq(0, 69, by = 5), 70, 80, 101)
#' age_pyramid <- data.frame(
#'   from = 0:101, weight = ifelse(0:101 < 65, 1, .99^(0:101-64))
#' )
#' age_pyramid$weight[102] <- 0
#' # flat age distribution, then 1% annual deaths, no one lives past 101
#'
#' alembic_dt <- alembic(ifr_levin, age_pyramid, age_limits, 0:101)
#'
#' ifr_blend <- blend(alembic_dt)
#' # the actual function
#' plot(
#'   60:100, ifr_levin(60:100),
#'   xlab = "age (years)", ylab = "IFR", type = "l"
#' )
#' # the properly aggregated blocks
#' lines(
#'   age_limits, c(ifr_blend$value, tail(ifr_blend$value, 1)),
#'   type = "s", col = "dodgerblue"
#' )
#' # naively aggregated blocks
#' ifr_naive <- ifr_levin(head(age_limits, -1) + diff(age_limits)/2)
#' lines(
#'   age_limits, c(ifr_naive, tail(ifr_naive, 1)),
#'   type = "s", col = "firebrick"
#' )
#' # properly aggregated, but not accounting for age distribution
#' bad_alembic_dt <- alembic(
#'   ifr_levin,
#'   within(age_pyramid, weight <- c(rep(1, 101), 0)), age_limits, 0:101
#' )
#' ifr_unif <- blend(bad_alembic_dt)
#' lines(
#'   age_limits, c(ifr_unif$value, tail(ifr_unif$value, 1)),
#'   type = "s", col = "darkgreen"
#' )
#' @export
blend <- function(
  alembic_dt
) {
  # creates appropriate parameter discretization for model partitions:
  # sum of all the mixing partition weights, divided by populations for each
  # model partition
  # weight i = integral_{c i}^{c i + 1} parameter(x)*rho(x) dx
  # relpop i = integral_{c i}^{c i + 1} rho(x) dx
  # i.e., the weighted average of the parameter
  return(alembic_dt[, .(value = sum(weight) / sum(relpop)), keyby = model_partition])
}

#' @title Distill Outcomes
#'
#' @description
#' `distill` takes a low-age resolution outcome, for example deaths,
#' and proportionally distributes that outcome into a higher age resolution for
#' use in subsequent analyses like years-life-lost style calculations.
#'
#' @inheritParams blend
#'
```

```r
#' @param outcomes_dt a long-format `data.frame` with a column either named
#' `from` or `model_from` and a column `value` (other columns will be silently
#' ignored)
#'
#' @param groupcol a string, the name of the outcome model group column. The
#' `outcomes_dt[[groupcol]]` column must match the `model_partition` lower
#' bounds, as provided when constructing the `alembic_dt` with [alembic()].
#'
#' @details
#' When the `value` column is re-calculated, note that it will aggregate all
#' rows with matching `groupcol` entries in `outcomes_dt`. If you need to group
#' by other features in your input data (e.g. if you need to distill outcomes
#' across multiple simulation outputs or at multiple time points), that has to
#' be done by external grouping then calling `distill()`.
#'
#'
#' @return a `data.frame`, with `output_partition` and recalculated `value` column
#'
#' @import data.table
#' @export
#' @examplesIf require(data.table)
#'
#' ifr_levin <- function(age_in_years) {
#'   (10^(-3.27 + 0.0524 * age_in_years))/100
#' }
#'
#' age_limits <- c(seq(0, 69, by = 5), 70, 80, 101)
#' age_pyramid <- data.frame(
#'   from = 0:101, weight = ifelse(0:101 < 65, 1, .99^(0:101-64))
#' )
#' age_pyramid$weight[102] <- 0
#' # flat age distribution, then 1% annual deaths, no one lives past 101
#'
#' alembic_dt <- alembic(ifr_levin, age_pyramid, age_limits, 0:101)
#'
#' results <- data.frame(model_partition = head(age_limits, -1))
#' results$value <- 10
#' distill(alembic_dt, results)
distill <- function(
  alembic_dt, outcomes_dt, groupcol = names(outcomes_dt)[1]
) {

  # compute the relative contribution of model partition j to output partition i
  # which is weight i / sum of all the weights in j
  mapping <- alembic_dt[, .(
    output_partition, model_fraction = weight / sum(weight)
    ), keyby = model_partition
  ]

  # these next steps deal with error handling for the outcomes that need
  # partition
  setnames(setDT(outcomes_dt), groupcol, "model_partition")
  uniqgroups <- outcomes_dt[, unique(model_partition)]
  uniqalemb <- alembic_dt[, unique(model_partition)]

  if (length(setdiff(uniqgroups, uniqalemb))) {
    stop(sprintf(
      "`outcomes_dt[[%s]] has groups not present in `alembic_dt$model_partition`: %s",
      groupcol,
      toString(setdiff(uniqgroups, uniqalemb))
```

```r
    ))
  }
  if (length(setdiff(uniqalemb, uniqgroups))) {
    warning(sprintf(
      "`outcomes_dt[[%s]] does not cover the groups present in `alembic_dt$model_partition`: missing %s",
      groupcol,
      toString(setdiff(uniqalemb, uniqgroups))
    ))
  }

  # if the outcomes have the proper shape, then join the mapping weights, then
  # for each output partition, sum the associated model outcomes according to
  # weighted contribution
  return(outcomes_dt[mapping, on = .(model_partition)][,
    .(value = sum(value * model_fraction)),
    by = output_partition
  ])

}
```

Section 2. Documentation for *alembic()*

`alembic`	*Create the Blending and Distilling Object*

Description

Based on model and output partitions, create a mixing partition and associated weights. That table of mixing values can be used to properly discretize a continuously varying (or otherwise high resolution) parameter to a relatively low resolution compartmental stratification, and then subsequently allocate the low-resolution model outcomes into the most likely high-resolution output partitions.

Usage

```r
alembic(
  f_param,
  f_pop,
  model_partition,
  output_partition,
  pars_interp_opts = interpolate_opts(fun = stats::splinefun, kind = "point", method =
    "natural"),
  pop_interp_opts = interpolate_opts(fun = stats::approxfun, kind = "integral", method =
    "constant", yleft = 0, yright = 0)
)
```

Arguments

`f_param`
: a function, `f(x)` which transforms the feature (e.g. age), to yield the parameter values. Alternatively, a `data.frame` where the first column is the feature and the second is the parameter; see `xy.coords()` for details. If the latter, combined with `pars_interp_opts` to create a parameter function.

`f_pop`
: like `f_param`, either a density function (though it does not have to integrate to 1 like a pdf) or a `data.frame` of values. If the latter, it is treated as a series of populations within intervals, and then interpolated with `pop_interp_opts` to create a density function.

`model_partition`
: a numeric vector of cut points, which define the partitioning that will be used in the model; must be length > 1

`output_partition`
: the partition of the underlying feature; must be length > 1

pars_interp_opts
: a list, minimally with an element `fun`, corresponding to an interpolation function. Defaults to `splinefun()` "natural" interpolation.

pop_interp_opts
: like `pars_interp_opts`, but for density. Defaults to `approxfun()` "constant" interpolation.

Details

The `alembic` function creates a mixing table, which governs the conversion between model and output partitions. The mixing table a `data.table()` where each row corresponds to a mixing partition c_i, which is the union of the model and output partitions - i.e. each unique boundary is included. Within each row, there is a `weight` and `relpop` entry, corresponding to

$$\text{weight}_i = \int_{c_i} f(x)\rho(x)\mathrm{d}x$$

$$\text{relpop}_i = \int_{c_i} \rho(x)\mathrm{d}x$$

where $f(x)$ corresponds to the `f_param` argument and $\rho(x)$ corresponds to the `f_pop` argument.

This mixing table is used in the `blend()` and `distill()` functions.

When `blend`ing, the appropriately weighted parameter for a model partition is the sum of weight_i divided by the relpop_i associated with mixing partition(s) in that model partition. This corresponds to the properly, population weighted average of that parameter over the partition.

When `distill`ing, model outcomes associated with weighted parameter from partition j are distributed to the output partition i by the sum of weights in mixing partitions in both j and i divided by the total weight in j. This corresponds to proportional allocation according to Bayes rule: the outcome in the model partition was relatively more likely in the higher weight mixing partition.

Value

a `data.table` with columns: `model_partition`, `output_partition`, `weight` and `relpop`. The first two columns identify partition lower bounds, for both the model and output, the other values are associated with; the combination of `model_partition` and `output_partition` forms a unique identifier, but individually they may appear multiple times. Generally, this object is only useful as an input to the `blend()` and `distill()` tools.

See Also

`blend()`

`distill()`

Examples

```
ifr_levin <- function(age_in_years) {
  (10^(-3.27 + 0.0524 * age_in_years))/100
}
age_limits <- c(seq(0, 69, by = 5), 70, 80, 101)
age_pyramid <- data.frame(
  from = 0:101, weight = ifelse(0:101 < 65, 1, .99^(0:101-64))
)
age_pyramid$weight[102] <- 0
# flat age distribution, then 1% annual deaths, no one lives past 101
ifr_alembic <- alembic(ifr_levin, age_pyramid, age_limits, 0:101)
```
