# Supplementary material for: paramix: An R package for parameter discretisation in compartmental models, with application to calculating years of life lost
Source: PLoS Comput Biol. 2025 Sep 8;21(9):e1013420. doi: 10.1371/journal.pcbi.1013420 (PMC12425178; doi:10.1371/journal.pcbi.1013420)
Supplement: Attachment — Submitted filename: Paramix response to reviewers.pdf [file pcbi.1013420.s002.pdf]

9 June 2025

To the Editor:

We thank the reviewers for the helpful comments which have improved the manuscript. We have attended to all of the comments and provide a point-by-point discussion of additions and modifications below.

In brief, we have made substantial clarifications in the mathematical description of what the tool does. We have additionally updated the package itself to expand the documentation adding these details, and reproduced that documentation as a supplement.

We look forward to the next steps.

Sincerely,

Carl Pearson
On behalf of the authors

Reviewer Comments

Reviewer #1: The paper is very well-written, easy to read, and is potentially relevant, with practical applications. I only have a few minor comments:

- Within integrals, the d in dx should not be italicized.

Thank you for highlighting this. We have now corrected the italicisation in the integrals.

- I would bring the definition of mixing table (L62-64, Fig 2) before the introduction of the workflow (L53-60). Now the workflow refers to the mixing table, but the reader can't know what does it mean, as it is introduced only later.

Thank you for this suggestion. We have forced the figure layout to occur after where this term is introduced in the text.

- I would give a unit of measurement for ρ.

rho is generally population density by feature. In our example, the feature is age, but more broadly it corresponds to whatever feature the model space is partitioning on. We have adjusted the text to highlight this, now reading as follows (line 74):

> *$\rho(x) > 0$ is the population density per feature of interest, with $\int_C \rho(x) \text{d}x = 1$.*

- Equation (4) is unclear to me: What does "in group [e, f)" mean...? It should be properly defined.

Thank you for highlighting that these terms needed formal definitions. We have rewritten this section to clarify model versus output versus mixing partitions, and how they relate to each other for calculating weightings.

- Equation (5) is unclear to me. "outcome" is never defined, and it is used somewhat inconsistently: on the left-hand-side we have $outcomes_x$, on the right hand side we have $outcomes_x^y$. These should be properly defined.

We have clarified this to provide example outcomes (such as hospitalisations or deaths), and to simplify the mathematical notation. The clarification reads as such (lines 84-87):

> *The distill() function distributes model outcomes (for example, estimated hospitalisations or deaths) to the output partition b_i from intersecting model partition(s) in A, i.e. where either the lower or upper bound of an a_j is within b_i: a_j \le b_i < a_{j+1} or b_i < a_{j+n} \le b_{i+1}.*

- I'd add a citation to SEIR, and age-stratified SEIR models.

Thank you for this suggestion; we have added a reference to Anderson and May 1991, *Infectious Diseases of Humans: Dynamics and Control*, where SEIR models and age-stratified SEIR models are first introduced (line 127).

- "To ensure comparability of the vaccination programmes, we assumed that enough vaccines to vaccinate 75% of over 65s could instead be allocated to different age groups, which typically entails lower coverage but otherwise never approaches 100% coverage." (L102-104) I don't really understand this sentence, could you please elaborate?

We have clarified this text, which now reads (lines 120-124):

> *To ensure comparability of the vaccination programmes, we assumed that a fixed number of doses corresponding to vaccinating 75% of over 65s was available. For each scenario, we allocated those doses to the target age group; for the under 65 targets, this typically yields lower than 75% coverage.*

- Fig 4b according to the figure caption ("Age-specific remaining life expectancy") is missing from the figure itself. (Thus the remaining ones have wrong letter.) It is also referred to in the text, despite not appearing in the figure.

Thank you for pointing out this error. We have corrected this omission, and removed these references to the subfigure in the Figure 4 caption and main text. The caption now reads as such:

> *Fig 4. a. Population age distribution. b. Age-specific infection fatality ratio, and the results of aggregating into broad age groups using four different methods, for both a flu-like and COVID-19-like infection. c. Incidence of flu-like and COVID-19-like infections, under either no vaccination program or vaccination of specific age groups. Subpanels on the left use the demography of a low- and middle-income country (LMIC); subpanels on the right use the demography of a high-income country (HIC).*

- It is strange that Figure 4c (actually 4b, see above) is referred before 4a in the text.

We have moved the text referring to Fig 4b (introducing the infection fatality ratios) to be below the text referring to Fig 4a (which introduces the different age structures).

- "and approaches 3 and 4 produced similar estimates of deaths averted" (L170) I don't understand this, there are only 3 approaches for aggregation.

This was a reference to previous analyses that we dropped prior to submission; thank you for pointing this out - we have removed this sentence.

Reviewer #2: Goodfellow et al. present an R package to bin parameters that vary as a function of a continuous variable (e.g., mortality rate as a function of age) into a small number of discrete values (e.g., mortality in young, mortality in middle-aged, mortality in elderly). They make a clear argument against using a value at the midpoint of each discrete bin and instead point out that a more statistically justifiable approach would be to take each parameter's expectation over the continuous variable's probability across each bin. While this R package addresses a clear problem, insufficient details are included to fully understand the approach without looking at the source code.

I suggest either going into more formal detail about the maths underlying the methods (more like a methods paper rather than a software paper) or by explicitly working through an example using the R functions in this package (more like a R vignette). For example,

We appreciate this feedback and agree that more clarity in methods would better serve readers. We have generally adjusted the mathematical description to further clarify how the calculations work. We have added additional signposting to elements of the package, which does include a simpler example worked as vignette as well as the full analysis presented in this work as an artifact available from the inst/ folder.

We have expanded both the help files and the internal code documentation in the package to explain how the code corresponds to the mathematical steps; this documentation is available to any user when they use the R help system or they print the function body. For readers attempting to understand the processes without actually installing the package or looking at the code, we have curated the relevant portions into a supplement.

- Equations 1-5 reference bounds a,b,c,d,e,f without explicitly defining;

Thank you for this point. We have generally revised the mathematical description for clarity, and these bounds are now captured in terms of the model versus output versus mixing partition boundaries. The introduction of these partitions now reads as follows (lines 68-71):

> *Let A = {ai} be the (strictly increasing) boundaries for the model partition, B = {bi} the boundaries for the output partition, and C = {ci} their union, the mixing partition (Fig 2). For brevity, we will denote partitions [xi, xi+1) by their lower bounds, xi (with x as a, b, or c as appropriate).*

- The term "mixing partition" is used without a definition;

We have now ensured that the mixing partition is defined when introduced; it is the union of the model and output partitions. Its definition reads as follows (lines 61-65):

> *The alembic() function uses the model and output partitions, e.g. the age groups for the compartments and the age resolution for outcomes, to create a mixing partition. This mixing partition, the union of model age bounds and output age bounds, sets the intervals for calculating weighted parameter integrals (hereafter, weights) and populations (Fig 2).*

- The parameters to the SEIR model are described only in the figure caption but not explicitly referenced in Table 1 which gives numerical values; etc.

Thank you for highlighting this omission. We have now added these definitions to Table 1. We have also added a force of infection equation to link the transmissibility parameter referenced in Table 1 to the model diagram in Figure 3.

Specific points

• fig 1: purple circles are labeled as “user inputs,” but the circle at the bottom (“post-analysis e.g. YLLs”) seems more like an output. Maybe label purple as “inputs/outputs”.

Thank you for this suggestion; we have incorporated this and updated Figure 1.

• line 71: talking about “interpolation” here made me question “what type?” until the later section that says your package uses both linear and spline interpolation. Would be better to give this information when first mention it!

Thank you for this point; we have moved this information to the first mention of interpolation, which now reads as (lines 75-77):

> *Internally, paramix handles converting from tabular input into functional forms using base R interpolation functions (splinefun() for parameters and approxfun() for density), but users can provide a custom interpolation function.*

• line 87: the last word on this line (“data.tables”) is all in italics, which implies the name of this class has an “s” on the end. A better phrasing might be “\emph{paramix} returns \emph{data.table} objects”.

We agree, and have corrected this. This sentence now reads (lines 104-106):

> *The paramix package inputs can be either functions or data.frame objects (or any type that extends data.frame, such as data.table or tibble [3, 4]), though paramix returns data.table objects.*

• fig 4: The caption needs more detail and should explicitly state that all subpanels on the left follow the LMIC-like distribution and all subpanels on the right follow the HIC-like distribution (also “LMIC” and “HIC” should be defined in the caption, not just the main text). Further, this figure seems to have been changed from an earlier version – some places refer to four panels (e.g., in the figure caption, and in text at bottom of page 5) and in other places three panels (e.g., the figure itself and in text on page 8).

We have incorporated these suggestions. The legend now reads:

> *Fig 4. a. Population age distribution. b. Age-specific infection fatality ratio, and the results of aggregating into broad age groups using four different methods, for both a flu-like and COVID-19-like infection. c. Incidence of flu-like and COVID-19-like infections, under either no vaccination program or vaccination of specific age groups. Subpanels on the left use the demography of a low- and middle-income country (LMIC); subpanels on the right use the demography of a high-income country (HIC).*

• fig 5 & 6: interpretation would be easier if the four subpanels in these figures were transposed such that the layout matches fig 4 (i.e., LMIC-like is on left, HIC is on right, Flu-like is on top, COVID-like is on bottom).

Thank you for this point. We have incorporated these suggestions, and updated Figures 5 and 6.
